# Protein Intake and Sarcopenia in Older Adults: A Systematic Review and Meta-Analysis

**DOI:** 10.3390/ijerph19148718

**Published:** 2022-07-18

**Authors:** Hélio José Coelho-Junior, Riccardo Calvani, Domenico Azzolino, Anna Picca, Matteo Tosato, Francesco Landi, Matteo Cesari, Emanuele Marzetti

**Affiliations:** 1 Department of Geriatrics and Orthopedics, Università Cattolica del Sacro Cuore, L.go F. Vito 1, 00168 Rome, Italy; francesco.landi@unicatt.it (F.L.); emanuele.marzetti@policlinicogemelli.it (E.M.); 2 Fondazione Policlinico Universitario “Agostino Gemelli” IRCCS, L.go A. Gemelli 8, 00168 Rome, Italy; riccardo.calvani@policlinicogemelli.it (R.C.); anna.picca@policlinicogemelli.it (A.P.); matteo.tosato@policlinicogemelli.it (M.T.); 3 Department of Clinical and Community Sciences, Università di Milano, Via Festa del Perdono 7, 20122 Milan, Italy; matteo.cesari@unimi.it; 4 Geriatric Unit, IRCCS Istituti Clinici Scientifici Maugeri, Via Camaldoli 64, 20138 Milan, Italy

**Keywords:** nutrition, anorexia, physical function, walking speed, muscle strength, dynapenia, frailty, elderly

## Abstract

Background: The present systematic review and meta-analysis investigated the cross-sectional and longitudinal associations between protein intake and sarcopenia in older adults. Methods: Observational studies that investigated the association between protein intake and sarcopenia as the primary or secondary outcome in people aged 60 years and older were included. Studies published in languages other than English, Italian, Portuguese, and Spanish were excluded. Studies were retrieved from MEDLINE, SCOPUS, EMBASE, CINAHL, AgeLine, and Food Science Source databases through January 31, 2022. A pooled effect size was calculated based on standard mean differences. Results: Five cross-sectional studies, one longitudinal study, and one case-control study that investigated 3353 community-dwelling older adults with a mean age of approximately 73 years were included. The meta-analysis of four studies indicated that older adults with sarcopenia consumed significantly less protein than their peers with no sarcopenia. Conclusions: Results of the present study suggest that an inadequate protein intake might be associated with sarcopenia in older adults.

## 1. Introduction

Sarcopenia is a neuromuscular disease characterized by muscle atrophy, dynapenia, and loss of physical function [1,2,3,4,5]. The overall prevalence of sarcopenia might reach up to 86.5% in adults depending on the definition used and the setting of evaluation, and is especially high in the older population [6]. This scenario deserves concern, given that the progression of sarcopenia is associated with the incidence of numerous negative events, including malnutrition, anorexia, physical inactivity, metabolic and osteoarticular disorders, cognitive impairment, falls, depressive symptoms, and death [7,8]. As such, sarcopenia is recognized as a public health problem and the identification of potential strategies to prevent its development and progression is a priority [1,2].

Nutrition is a modifiable lifestyle factor that may be harnessed to foster active and healthy aging [9,10]. In particular, a protein consumption higher than the current recommended dietary allowance (RDA, 0.8 g/kg/day) is proposed as a strategy to preserve muscle mass and physical function in advanced age [11,12,13,14]. This recommendation is based on the fact that the aged muscle requires a greater amount of amino acids (AAs) to maximally stimulate muscle protein synthesis (MPS) in response to hyperaminoacidemia, a phenomenon known as anabolic resistance [15,16,17,18,19,20]. The failure to properly stimulate MPS predisposes to gradual loss of muscle mass, mainly of type II muscle fibers, which impacts muscle strength generation and physical function [21,22]. Although it is commonly believed that an adequate protein intake could prevent the development of sarcopenia or at least attenuate its progression [23,24,25], findings on the matter are inconclusive.

To provide an up-to-date and comprehensive appraisal of the topic, we conducted a systematic review and meta-analysis of cross-sectional and longitudinal studies that explored the relationship between protein intake and sarcopenia in older adults.

## 2. Materials and Methods

This is a systematic review and meta-analysis of observational studies that investigated cross-sectional and longitudinal associations between protein intake and sarcopenia. The study was fully performed by investigators and no librarian was part of the team. The study complies with the criteria of the Meta-analysis of Observational Studies in Epidemiology (MOOSE) guidelines [26] and the Cochrane Handbook for Systematic Reviews and Interventions [27].

### 2.1. Eligibility Criteria

Inclusion criteria were: (1) observational studies (e.g., case-control, cross-sectional, cohort longitudinal studies) that investigated the association between protein intake and sarcopenia; (2) participants aged 60 years or older; (3) sarcopenia identified according to the presence of muscle atrophy plus dynapenia, low muscle power, physical dysfunction, reduced mobility, and/or low scores on batteries of physical performance tests (e.g., Short Physical Performance Battery (SPPB)); (4) published studies in English, Italian, Portuguese, or Spanish language.

To be included in the meta-analysis of cross-sectional studies, investigations should provide mean and standard deviation (SD) of case (i.e., high protein intake (HPI)) and control groups (i.e., low protein intake, (LPI)), or at least two groups divided according to protein consumption and the sample size of each group, or Pearson’s correlation coefficient (r)/Betas (β)/odds ratio (OR) values for the association between protein intake and sarcopenia. For the meta-analysis of longitudinal studies, investigations should provide the number of participants, β, OR, hazard ratio, and/or risk ratio for the development of sarcopenia according to protein consumption levels. We excluded randomized controlled trials, quasi-experimental, cross-over, and preclinical studies, as well as investigations that examined the effects of nutritional interventions alone or combined with other interventions (e.g., physical exercise) on sarcopenia. Studies that enrolled participants with gastrointestinal and/or renal diseases, anorexia, cancer, or any condition that may directly impair protein metabolism (e.g., maple syrup urine disease, tyrosinemia) were also excluded.

### 2.2. Search Strategy and Selection Criteria

Studies published on or before 31 January 2022 were retrieved from the following six electronic databases by one investigator: (1) MEDLINE (PubMed interface); (2) SCOPUS (Elsevier interface); (3) EMBASE (OVID interface), (4) CINAHL (EBSCO interface); (5) AgeLine (EBSCO interface); and (6) Food Science Source (EBSCO interface). Further eligible articles were identified by checking the reference lists of retrieved articles. In addition, citation searches on key articles were performed in Google Scholar and ResearchGate. Initially, a search strategy was designed using keywords, MeSH terms, and free text words, such as "protein intake", "sarcopenia", and "older adults". Afterwards, keywords and subject headings were exhaustively combined using Boolean operators. The complete search strategy is shown in Supplementary Material S1.

### 2.3. Data Extraction, Quality Assessment, and Risk of Bias

Titles and abstracts of retrieved articles were screened for eligibility by two researchers (HJCJ, RC). The full text was consulted if the abstract did not provide enough information for final evaluation. Two reviewers (HJCJ, RC) extracted the coded variables (i.e., methodological quality, risk of bias, and characteristics of the studies) using a standardized coding form. A third researcher was consulted to solve disagreements (EM), if necessary. The quality of reporting for each study was performed by two researchers (HJCJ, RC) using the Quality Assessment Tool for Observational Cohort and Cross-Sectional of the National Institutes of Health [28]. This tool contains 14 questions that assess several aspects associated with the risk of bias, type I and type II errors, transparency, and confounding factors. The studies were positive for item 8 if they investigated protein sources and/or distribution. Items 6, 7, and 13 do not refer to cross-sectional studies and were removed from the quality analysis. The maximum scores for cross-sectional and prospective studies were 11 and 14, respectively. The agreement rate for quality assessment between reviewers was 98%.

### 2.4. Statistical Analysis

The meta-analysis was conducted using Revman 5.4.1 (Cochrane Collaboration, Copenhagen, Denmark). Effect sizes (ESs) were measured using means and SDs. Central and dispersion values were obtained from included studies or were calculated according to the Cochrane guidelines [27]. Specifically, medians were assumed as means when studies presented symmetrical data. SDs were calculated from confidence intervals (CIs) and standard errors (SEs), according to the following formulas:

SD1 = √N × (Upper limit − Lower limit)/3.92(1)

SD2 = SE × √N(2)

From the interquartile range, SDs were obtained according to the formulas proposed by Luo [29] and Shi [30]. A single pairwise comparison was created when multiple studies referred to the same database, using the formulas proposed by the Cochrane guidelines [27]. The pooled ES was calculated based on standard mean differences (SMDs), because studies used different tests and/or protocols to operationalize sarcopenia. Due to the variability of sample characteristics, a random-effect model was used to calculate the pooled ES. Additionally, the I^2^ index was classified as "might not be important" (0–40%), "may represent moderate heterogeneity" (30–60%), "may represent substantial heterogeneity" (50–90%), or "may represent considerable heterogeneity" (75–100%) [27]. Forest plots were used to illustrate the summary statistics and the variation (heterogeneity) across studies.

## 3. Results

### 3.1. Literature Search

**Figure 1:**
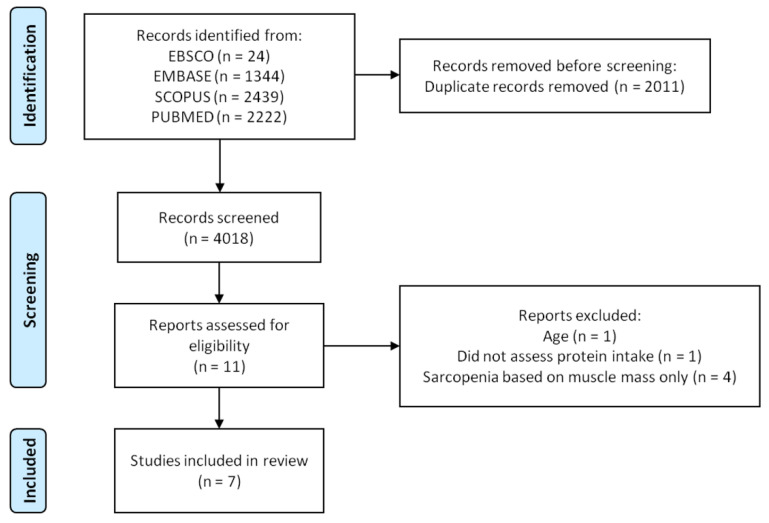
Flowchart of the study.

Six-thousand and twenty-nine records were identified through database and hand searches. Of these, 2011 were excluded based on duplicated data, and 4018 titles and abstracts were evaluated. Eleven articles were fully assessed for eligibility and four studies were excluded based on selection criteria (Supplementary Material S2). Seven articles were included in the investigation. The flowchart of the study is shown in Figure 1.

### 3.2. Characteristics of the Included Studies

The main characteristics of the included studies are shown in Table 1. Five cross-sectional studies [31,32,33,34,35], one longitudinal study [36], and one case-control study [37] that investigated 3353 community-dwelling older adults with a mean age of approximately 73 years from Australia, Belgium, Finland, India, and the Netherlands were included. One study [32] included participants from Italy, Poland, the Netherlands, and the United Kingdom. Nutritional habits were assessed using 24-h dietary recall, 3- and 7-day food records, diet history, and food frequency questionaries. Sarcopenia was operationalized according to the European Working Group on Sarcopenia in Older People (EWGSOP) [1], EWGSOP2 [2], and the Foundation for the National Institutes of Health (FNIH) sarcopenia project [5]. One study compared all three sarcopenia frameworks [34], and one study [32] diagnosed sarcopenia according to the presence of low skeletal muscle index (SMI) and SPPB score.

### 3.3. Quality Assessment

Quality assessment scores are shown in Supplementary Material S3. The overall score of cross-sectional studies [31,32,33,34,35] ranged from six to seven. All of the studies clearly stated the research question (item 1), specified the study population (item 2), recruited participants from the same or a similar population (item 4), clearly defined and used valid and reliable exposure (item 9) and outcome (item 11) measures. Four investigations reported a participation rate of eligible persons of at least 50% (item 3), two studies investigated different levels of exposure (item 8), and three investigations adjusted their results according to confounding parameters (item 14). No studies justified the sample size (item 5) or reported whether investigators were blinded to the exposure of participants (item 12).

The longitudinal study [36] had an overall score of 10. The study established the research question (item 1), specified the study population (item 2), investigated a study population with a participation rate of eligible persons of at least 50% (item 3), recruited participants from the same or a similar population (item 4), justified the sample size (item 5), measured the exposure of interest before the outcome being measured (item 6), used a timeframe sufficient to expect to see an association between exposure and outcome (item 7), clearly defined and used valid and reliable exposure (item 9) and outcome measures (item 11), and adjusted their results according to confounding parameters (item 14). The study did not investigate different levels of exposure (item 8), did not assess the exposure more than once (item 10), and did not report whether investigators were blinded to the exposure of participants (item 12).

The case-control study [37] had an overall score of eight. The study clearly stated the research question (item 1), specified the study population (item 2), recruited control and case participants from the same or a similar population (item 4), clearly defined the inclusion and exclusion criteria (item 5), clearly defined and differentiated cases from controls (item 6), selected the participants randomly from eligible candidates (item 7), used concurrent control (item 8), and clearly defined and used valid and reliable exposure (item 10). The study did not justify the sample size (item 3), did not confirm whether the exposure occurred before the development of sarcopenia (item 9), did not report whether assessors were blinded to case or control participants (item 11), and did not adjust results according to potential covariates (item 12).

### 3.4. Cross-Sectional Association between Protein Intake and Sarcopenia

**Figure 2:**
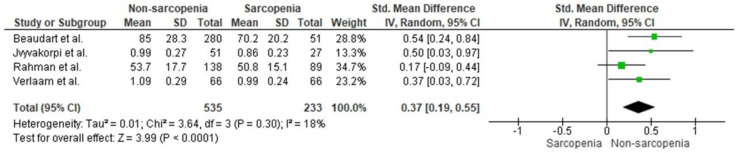
Standard mean differences of protein intake between older adults with and without sarcopenia [31,33,35,37].

The cross-sectional association between protein intake and sarcopenia is shown in Figure 2. Four studies were included in the pooled analysis [31,33,35,37]. Older adults with sarcopenia consumed significantly less protein than their non-sarcopenic counterparts (SMD = 0.37, 95% CI = 0.19–0.55, *p* < 0.0001). Heterogeneity was classified as "might not be important" (I^2^ = 18%, *p* = 0.30).

### 3.5. Cross-Sectional Association between Protein Sources and Sarcopenia

One study investigated the association between protein sources and sarcopenia [32]. Montiel-Rojas et al. [32] enrolled 986 older European adults and explored the association between protein sources and sarcopenia, diagnosed according to the presence of low SMI plus reduced handgrip strength. The authors found that the risk of sarcopenia was lower in those with greater protein consumption. In addition, the replacement of animal-derived proteins with an equal amount of plant-derived proteins was associated with a reduced risk of sarcopenia.

### 3.6. Longitudinal Associations between Protein Intake and Sarcopenia

One study investigated the longitudinal association between protein intake and incident sarcopenia [36]. Compared with older adults on a low-butter diet, those eating a traditional British diet (i.e., rich in butter, red meat, gravy, and potato) had an increased risk of sarcopenia over a 3-year follow-up even if protein intake was ≥1 g/kg of body weight (BW)/d. Results were similar when the HPI threshold was set at ≥0.8 g/kg of BW/d. However, the risk of incident sarcopenia at three years was no longer significant in the fully adjusted model.

## 4. Discussion

The present systematic review and meta-analysis investigated the association between protein intake and sarcopenia in older adults. The pooled analysis of cross-sectional studies indicated that older adults with sarcopenia have a lower intake of proteins compared with non-sarcopenic peers. Two additional potentially important results were observed. First, the consumption of plant-based protein was cross-sectionally associated with a low prevalence of sarcopenia. Second, older adults on a high-fat/high-energy diet may be at high risk of sarcopenia even if their protein intake is greater than the RDA.

HPI has long been considered to be a modifiable lifestyle factor that might potentially counteract sarcopenia [23,24,25]. This assumption is based on the effects of AAs on muscle protein metabolism. Muscle mass is regulated by a dynamic equilibrium between MPS and muscle protein breakdown (MPB) [38,39,40,41,42]. Adequate protein ingestion is expected to increase AA availability and stimulate sarcoplasmic and myofibrillar protein synthesis by activating the mammalian target of rapamycin (mTOR) and its downstream targets [38,39,40,41,42]. However, the aged muscle frequently shows anabolic resistance, a state of submaximal MPS in response to hyperaminoacidemia, suggesting that greater amounts of protein are required to properly stimulate muscle anabolism in older adults [15,16,17,18,19,20].

If the anabolic resistance is not overcome through the diet, an imbalance in muscle metabolism in favor of MPB might occur, promoting muscle loss [43]. Muscle atrophy occurs preferably in type II muscle fibers [21,22,44], those that contract faster and have a greater capacity to generate tension [21,22]. Hence, it may be expected that older adults with HPI might experience less muscle atrophy and neuromuscular dysfunction.

However, such a view is not supported by the only longitudinal investigation included in the present study. Granic et al. [36] observed that older adults on a traditional British diet and with a protein intake ≥1 g/kg of BW/d had an increased risk of developing sarcopenia compared with those on a low-butter diet during three years of follow-up. These findings have some possible explanations.

Protein quality refers to the anabolic response elicited by protein sources [45]. Numerous studies found that animal-based proteins produced greater muscular anabolism than plant-based proteins [40,46,47]. These divergent anabolic responses are attributed to differences in digestion and absorption rates, and branched-chain AA (BCAA) content [45,48]. Indeed, animal proteins are characterized by digestibility rates higher than 90%, whereas digestibility rates barely reache 50% with plant proteins [45,48]. Furthermore, animal proteins have a greater content of BCAAs in comparison to plant-based proteins [14,45]. Such data are important because BCAAs, and mainly leucine, are considered to be major stimulators of MPS [42,49,50].

Hence, it is possible that older adults with HPI who developed sarcopenia had a protein consumption mostly based on plant sources, providing an insufficient supply of AAs to properly stimulate MPS. Although this hypothesis offers a reasonable explanation for the report by Granic et al. [36], other investigations found that a high intake of plant-based proteins was associated with faster walking speed [51] and lower prevalence of frailty [52]. Experts in the field interpreted these findings as the indication that an adequate intake of vegetable proteins may also properly stimulate muscle anabolism [14]. Another possible explanation to the findings by Granic et al. [36] is that the traditional British diet is characterized by a high intake of fat and energy. Such dietary regimes are associated with an increased risk of obesity which, in turn, promotes the development of insulin resistance, oxidative stress, low-grade systemic inflammation, and hormonal changes [53]. All of these factors play a role in the pathophysiology of sarcopenia. Finally, results by Granic et al. [36] were not controlled for many covariables that might impact the association between protein intake and sarcopenia, including the practice of physical exercise [54,55], the presence of frailty [56], and oral health [57].

Only one study investigated the association between protein sources and sarcopenia [32]. Montiel-Rojas et al. [32] observed that the consumption of plant protein was negatively associated with the presence of sarcopenia. Additional studies investigating the potential role of protein sources on the development of sarcopenia are warranted.

Our study has limitations that deserve discussion. First, all of the investigations included examined community-dwelling older adults, and extrapolations to hospitalized patients and people living in long-term institutions should be made with caution. Second, our pooled analysis was conducted to identify differences in means and SDs, given the limited number of studies that performed regression analyses. This indicates that results were not adjusted for numerous covariables. Third, substantial heterogeneity was observed in the way protein consumption data were presented (e.g., absolute, adjusted according to BW, percentage of calories). Fourth, the limited number of included studies did not allow meta-regression, dose-response, risk of bias, or “trim and fill” analysis to be conducted. Fifth, the findings on the longitudinal association between protein intake and those on protein sources with sarcopenia were based on one study each. Sixth, different studies were included in the cross-sectional and longitudinal analyses, which might produce divergent results. Seventh, although most studies used EWGSOP criteria to identify people with sarcopenia, different instruments, cutoff points, and other operational definitions of sarcopenia were also utilized. This aspect deserves concern because protein intake might be associated with each one of those variables, therefore influencing our results. In fact, vegetal protein has been associated with walking speed, but not with muscle strength [51,58]. Eighth, most of the investigations were conducted in Europe. Finally, no studies took into account the severity of sarcopenia.

Notwithstanding, our study provides directions for future investigations. The finding that sarcopenic older adults consumed significantly less protein than their non-sarcopenic counterparts partially supports the assumption that protein intake is associated with sarcopenia and encourages the conduct of large multicentric, cross-sectional and longitudinal studies to better explore the subject. Future investigations should take into consideration several nutritional and sarcopenia-related aspects that are still lacking in the literature, including differences between protein sources, diagnostic criteria for sarcopenia, and sociocultural factors. The impact of relevant covariables should also be explored. The lack of this information still limits extrapolations of the current findings to clinical practice.

## 5. Conclusions

Our pooled analysis indicate that older adults with sarcopenia consumed significantly less protein than their non-sarcopenic counterparts. These results were based on differences in means and SDs, given the lack of investigations that conducted regression analyses. One cross-sectional study noted that plant-based protein might be negatively associated with the prevalence of sarcopenia. On the other hand, a longitudinal study observed that older adults following a traditional British dietary pattern had an increased risk of sarcopenia even if protein intake was high. These findings suggest that more cross-sectional and longitudinal studies, with deeper statistical approaches and more comprehensive analyses of protein-related parameters are required to confirm and expand the current results.

## Figures and Tables

**Table 1:** Characteristics of the included studies.

Year	Author	Study Type	Follow-Up (Years)	Country	Sample Characteristics	Sample Size	Mean Age (Years)	Mean Daily Protein Intake	Dietary Intake Assessment Method	Sarcopenia Assessment Method
2017	Veerlan et al. [37]	Case-Control	—	Netherland	Community-dwelling older adults	132	~71	~73.9 g	3-d food record	(a) SMI and (b) SPPB
2019	Beaudart et al. [35]	Cross-sectional	—	Belgium	Community-dwelling older adults	331	74.8	~82.7 g	Food frequency questionnaire	EWGSOP
2020	Das et al. [34]	Cross-sectional	—	Australia	Community-dwelling older men	794	81.1	—	Diet history questionnaire	FNIH, EWGSOP, and EWGSOP2
2020	Granic et al. [36]	Longitudinal	3	United Kingdom	Community-dwelling older adults	757	85+	—	24-h dietary recall	EWGSOP
2020	Jyväkorpi et al. [33]	Cross-sectional	—	Finland	Community-dwelling older adults	126	~87.4	~0.93 g/kg BW	3-d food record	EWGSOP2
2020	Montiel-Rojas et al. [32]	Cross-sectional	—	Europe	Community-dwelling women	986	~71	—	7-d food record	EWGSOP2
2021	Rahman et al. [31]	Cross-sectional	—	Indian	Community-dwelling women	227	65.1	~52.2 g	Diet history	EWGSOP

BW= body weight; EWGSOP = European Working Group on Sarcopenia in Older People; FNIH, Foundation for the National Institutes of Health; SMI = Skeletal muscle index; SPPB = Short Physical Performance Battery.

## Data Availability

Data are available in the manuscript.

## Supplementary Materials

The following supporting information can be downloaded at: https://www.mdpi.com/article/10.3390/ijerph19148718/s1, Supplementary Material S1: Search strategy, Supplementary Material S2: Reasons for study exclusion, Supplementary Material S3: Quality analysis.
