# Supplementary material for: Protein Intake and Sarcopenia in Older Adults: A Systematic Review and Meta-Analysis
Source: Int J Environ Res Public Health. 2022 Jul 18;19(14):8718. doi: 10.3390/ijerph19148718 (PMC9320473; doi:10.3390/ijerph19148718)
Supplement: Supplementary file 1 [file ijerph-19-08718-s001.zip › ijerph-1777093-supplementary.pdf]

Supplementary Material S1: Search strategy

PUBMED*

1. Protein intake AND Sarcopenia [MESH] AND Aged [MESH]
2. Protein intake AND Sarcopenia [MESH] AND Older Adults
3. Protein intake AND Sarcopenia [MESH] AND Elderly
4. Protein intake AND Sarcopenia [MESH] AND Seniors
5. Protein consumption AND Sarcopenia [MESH] AND Aged [MESH]
6. Protein consumption AND Sarcopenia [MESH] AND Older Adults
7. Protein consumption AND Sarcopenia [MESH] AND Elderly
8. Protein consumption AND Sarcopenia [MESH] AND Seniors

*The search was conducted using terms as MESH and free words.

EMBASE*

1. Protein intake AND Sarcopenia AND Aged
2. Protein intake AND Sarcopenia AND Older Adults
3. Protein intake AND Sarcopenia AND Elderly

*All searchers were conducted using the filter *Title, Abstract, Author Keywords*

SCOPUS*

1. Protein intake AND Sarcopenia AND Aged
2. Protein intake AND Sarcopenia AND Older Adults
3. Protein intake AND Sarcopenia AND Elderly
4. Protein consumption AND Sarcopenia AND Aged
5. Protein consumption AND Sarcopenia AND Older Adults
6. Protein consumption AND Sarcopenia AND Elderly

*All searchers were conducted using the filter *Article title, Abstract, Keywords*

EBSCO (AgeLine, CINAHL, Food Science Source)

1. Protein intake AND Sarcopenia AND Aged
2. Protein intake AND Sarcopenia AND Older Adults
3. Protein intake AND Sarcopenia AND Elderly
4. Protein intake AND Sarcopenia AND Senior
5. Protein intake AND Sarcopenia AND Geriatrics
6. Protein consumption AND Sarcopenia AND Aged
7. Protein consumption AND Sarcopenia AND Older Adults
8. Protein consumption AND Sarcopenia AND Elderly
9. Protein consumption AND Sarcopenia AND Senior
10. Protein consumption AND Sarcopenia AND Geriatrics

Supplementary Material S2: Reasons for study exclusion

Age

1. Celis-Morales, C.; Petermann-Rocha, F.; Chen, M.; Gray, S.R.; Ho, F.K.; Pell, J.P. Factors associated with sarcopenia: A cross-sectional analysis using UK Biobank. *Maturitas* **2020**, *133*, 60–67. https://doi.org/doi: 10.1016/j.maturitas.2020.01.004.

Did not assess protein intake

2. Li, C.; Kang, B.; Zhang, T.; Gu, H.; Song, P.; Chen, J.; Wang, X.; Xu, B.; Zhao, W.; Zhang, J. Dietary Pattern and Dietary Energy from Fat Associated with Sarcopenia in Community-Dwelling Older Chinese People: A Cross-Sectional Study in Three Regions of China. *Nutrients* **2020**, *12*, 3689. https://doi.org/10.3390/nu12123689.

Sarcopenia based on muscle mass only

3. Hong, J.; Shin, W.K.; Lee, J.W.; Kim, Y. Relationship Between Protein Intake and Sarcopenia in the Elderly with Nonalcoholic Fatty Liver Disease Based on the Fourth and Fifth Korea National Health and Nutrition Examination Survey. *Metab. Syndr. Relat. Disord.* **2021**, *19*, 452–459. https://doi.org/10.1089/met.2021.0011.
4. Oh, C.; Jeon, B.H.; Storm, S.N.R.; Jho, S.; No, J.K. The most effective factors to offset sarcopenia and obesity in the older Korean: Physical activity, vitamin D, and protein intake. *Nutrition* **2017**, *33*, 169–173. https://doi.org/10.1016/j.nut.2016.06.004.

Supplementary Material S3: Quality analysis

							Cross-sectional studies								
**Authors**	**1**	**2**	**3**	**4**	**5**	**6**	**7**	**8**	**9**	**10**	**11**	**12**	**13**	**14**	**Overall score (0/11)**
Beaudart et al. [35]	Y	Y	Y	Y	N	NA	NA	N	Y	NA	Y	NR	NA	Y	7
Das et al. [34]	Y	Y	Y	Y	N	NA	NA	N	Y	NA	Y	NR	NA	Y	7
Jyväkorpi et al. [33]	Y	Y	Y	Y	N	NA	NA	Y	Y	NA	Y	NR	NA	N	7
Montiel-Rojas et al. [32]	Y	Y	Y	Y	N	NA	NA	Y	Y	NA	Y	NR	NA	N	7
Rahman et al. [31]	Y	Y	N	Y	N	NA	NA	N	Y	NA	Y	NR	NA	Y	6
							Longitudinal study								
Granic et al. [36]	Y	Y	Y	Y	Y	Y	Y	N	Y	N	Y	NR	N	Y	10
							Case-control study								
	**1**	**2**	**3**	**4**	**5**	**6**	**7**	**8**	**9**	**10**	**11**	**12**		**Overall score (0/11)**	
Veerlan et al. [37]	Y	Y	N	Y	Y	Y	Y	Y	N	Y	N	N		8	

Y= Yes; N= No; NA= Not applicable; NR= Not reported
